# What defines a great surgeon? A survey study confronting perspectives

**DOI:** 10.3389/fmed.2023.1210915

**Published:** 2023-06-28

**Authors:** Romain Luscan, Emeline Malheiro, Fiona Sisso, Sébastien Wartelle, Yann Parc, Brigitte Fauroux, Thierry Bégué, Hubert Johanet, Françoise Denoyelle, Erea-Noël Garabédian, François Simon

**Affiliations:** ^1^Université Paris Cité, Faculté de Médecine, Paris, France; ^2^Department of Paediatric Otolaryngology, AP-HP, Hôpital Necker-Enfants Malades, Paris, France; ^3^Department of Otolaryngology, Centre Hospitalier Intercommunal de Créteil, Créteil, France; ^4^Department of Paediatric Anaesthesiology, AP-HP, Hôpital Necker-Enfants Malades, Paris, France; ^5^Department of Paediatric Otolaryngology, Clinique Marcel Sembat, Boulogne-Billancourt, France; ^6^Paris Sorbonne University, Faculté de Médecine, Paris, France; ^7^Department of Digestive Surgery, AP-HP, Hôpital Saint Antoine, Paris, France; ^8^Pediatric Noninvasive Ventilation and Sleep Unit, AP-HP, Hôpital Necker Enfants-Malades, Paris, France; ^9^Université Paris Cité, EA 7330 VIFASOM (Vigilance Fatigue Sommeil et Santé Publique), Paris, France; ^10^Paris-Saclay University, Faculté de Médecine Paris-Saclay, Le Kremlin-Bicêtre, France; ^11^Department of Orthopaedic Surgery and Traumatology, AP-HP, Hôpital Antoine Béclère, Clamart, France; ^12^Department of General Surgery, Clinique Turin, Paris, France; ^13^Académie Nationale de Chirurgie, Paris, France

**Keywords:** surgeon, technical skills, non-technical skills, surgery, survey

## Abstract

**Background:**

The definition of a *great surgeon* is usually reported by surgeons themselves. The objective of the study was to define a multifaceted definition of a *great surgeon*, by confronting patients', healthcare workers', and surgeons' perspectives.

**Study design:**

An online open-ended questionnaire was created to identify three qualities and three shortcomings defining a *great surgeon*. Age, gender, and profession of respondents were collected. Responses with a similar meaning were combined into word groups and labeled within four themes: human qualities, technical surgical skills (TSS), non-technical skills (NTS), and knowledge. Multivariate analyses were conducted between themes and respondent characteristics.

**Results:**

Four thousand seven hundred and sixty qualities and 4,374 shortcomings were obtained from 1,620 respondents including 385 surgeons, 291 patients, 565 operating theater (OT) health professionals, and 379 non-OT health professionals. The main three qualities were dexterity (54% of respondents), meticulousness (18%), and empathy (18%). There was no significant difference between professional categories for TSS. Compared with surgeons, non-OT health professionals and patients put more emphasis on human qualities (29 vs. 39% and 42%, respectively, *p* < .001). OT health professionals referred more to NTS than surgeons (35 vs. 22%, *p* < 0.001). Knowledge was more important for surgeons (19%) than for all other professional categories (*p* < 0.001).

**Conclusions:**

This survey illustrates the multifaceted definition of a *great surgeon*. Even if dexterity is a major quality, human qualities are of paramount importance. Knowledge seems to be underestimated by non-surgeons, although it essential to understand the disease and preparing the patient and OT team for the procedure.

## Introduction

What defines a *great surgeon*? This question has obsessed generations of surgeons whose social status, training, knowledge, and skills have evolved remarkably over the centuries, from the first prehistoric surgeons trepanning skulls with flintstones to the modern-day technologically assisted surgeons. As Robert Liston's “fastest knife in the West End” in the pre-anesthesiology era when speed was flaunted rather than surgical outcomes, what might be expected of a *great surgeon* has substantially changed (1). The twentieth-century surgeon had to be “supremely confident, flamboyant, dogmatic and rich” while the importance of the surgical procedure could be judged by the size of the scar “big surgeon-big cut” (2). A great surgeon would also be bold, inventive (3, 4), as well as wise: “A good surgeon knows how to operate; a better surgeon knows when to operate but the best surgeon knows when not to operate” (2).

In the current era of patient-centered care and keyhole procedures, what makes a great twenty-first-century surgeon? The definition of a great surgeon will always be biased, based on personal experience, context, and relationship toward the surgical profession. The contemporary surgeon works as part of a larger healthcare team and must therefore reflect on his skills but also be aware of the patients' and colleagues' point of views.

Many papers on the topic can be found in the literature, mostly from a surgical point of view by experienced surgeons looking back on their career and colleagues, but theirs is not the only opinion to value (2, 5–9). In a modern large multispecialty operating theater, teams and colleagues are often quick to label surgeons (sometimes unfairly) as excellent or poor surgeons, most of the time based on their operative speed and human qualities with other professions. In a national or international setting, great surgeons are often considered as such when innovative and good at communicating their results. Finally for patients, reputation, empathy, and communication are important issues, but which characteristics stand out when a surgeon is being given a 5-star ranking online?

The objective of this study was to ask peers, colleagues, and patients, their definition of a *great surgeon*. The surgeons' main qualities and shortcomings were analyzed to understand how differently they may be perceived according to professional category, age, or sex.

## Methods

An online survey was distributed in February 2021 to four French medical societies' mailing lists: ENT, orthopedics, digestive surgery, and anesthesiology societies (*Société Française d'Oto-rhino-laryngologie, Société Française d'Orthopédie* and *Société Française de chirurgie Digestive, Société Française d'Anesthésie et Réanimation);* to the French Academy of Surgery's and Parisian Surgeons' Union's mailing-lists *(Académie Nationale de Chirurgie* and *Syndicat des chirurgiens des hôpitaux de Paris)*; to official French national surgical nurses' and nurse anesthesist's social network groups (i.e., private Facebook groups); to patients' social groups (non-healthcare respondents answered as patients); and finally the authors' personal or professional circles (i.e., healthcare professionals from their own Academic or non-Academic hospitals).

Participation was anonymous, and no ethics committee approval was required. It was not appropriate to involve patients or the public in the design, conduct, reporting, or dissemination plans of our research.

Respondents answered open-ended questions: “What do you think defines an excellent surgeon? Please indicate up to three key qualities you consider most important to be an excellent surgeon and conversely up to three key shortcomings a surgeon should not have.”

Age, gender, and profession were collected: surgeons, anesthesiologists, medical doctors who did not work in the operating theater (OT), surgical nurses, nurse anesthetists, other paramedical professions who do not work in the operating theater (non-OT), and finally patients.

### Data preparation for analysis

Participants' answers from the same lexical field or with similar meaning (single words or multiword expressions) were combined into word groups, and all words in a given group were changed to the most frequently used word within that group, to simplify data presentation and analysis. The word groups were then labeled within one of the four following themes: (1) human and moral qualities, (2) technical surgical skills (TSS), (3) non-technical skills (NTS) (10, 11), and (4) medical knowledge. Word groups and themes were determined manually by four independent investigators and discussed among authors until a consensus was reached.

For analysis purposes, respondents' professions were also grouped into four categories according to their daily interaction with surgeons: (1) surgeons, (2) operating-theater (OT) health professionals excluding surgeons (i.e., anesthesiologists, nurse anesthetists, and surgical nurses), (3) non-OT health professionals (i.e., non-OT medical doctors and non-OT paramedical professions), and (4) patients. Age categories were defined as younger than 35 years old, between 35 and 50, and older than 50.

### Statistical analysis

Univariate (χ2 or Fisher's test exact when appropriate) analyzed the relationship between response themes and professional categories on one hand, and qualities/shortcomings and respondents' profession on the other hand. Univariate and multivariate analyses (logistic regression) were performed to identify predictive factors of qualities' themes. Statistical analyses were performed using GraphPad Prism 9.0 (GraphPad software, San Diego, CA, USA). *P*-value < 0.05 was considered significant.

## Results

Respondents included 546 males and 1,074 females, totaling 1,620 subjects, mean age 43 ± 13 years (range 16–90). Professional categories were as follows: 385 surgeons, 291 patients, 565 OT health professionals [anesthesiologists (*n* = 53), surgical nurses (*n* = 245), nurse anesthetists (*n* = 267)], and 379 non-OT health professionals [non-OT medical doctors (*n* = 108), and non-OT paramedical professions (*n* = 271)].

### Overall view

**Figure 1:**
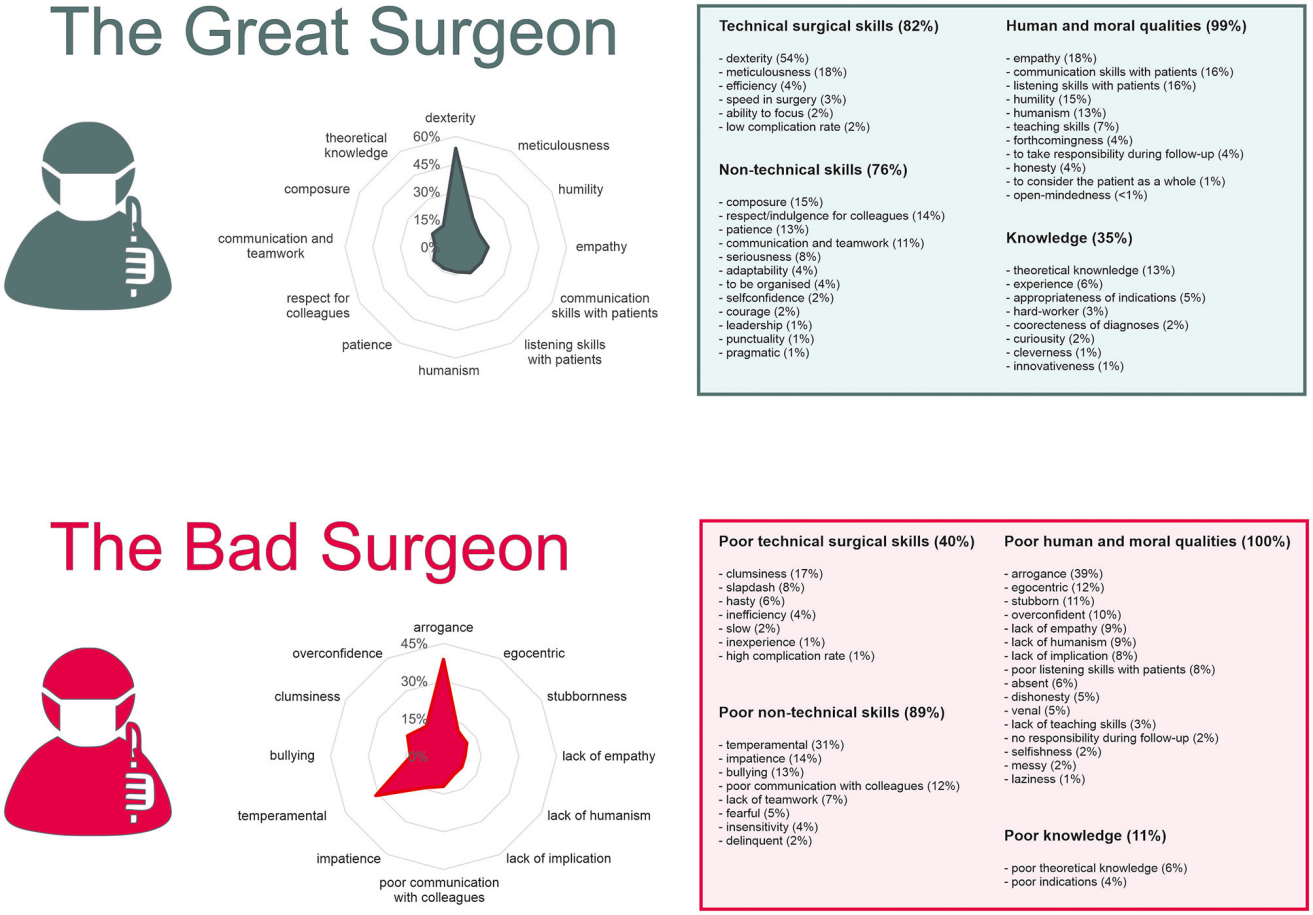
What makes a great surgeon? Overall results of the 1,620 respondents. Qualities (4,760 responses) are reported in the ≪ Great Surgeon ≫ section. Shortcomings (4,374 responses) are reported in the ≪ Bad Surgeon ≫ section. Radar graphs show how are distributed the first 12 characteristics (in proportion of respondents who have given those characteristics). Tables on the right report the themes and characteristics with proportion of respondents having answered that theme or characteristic.

A total of 4,760 qualities and 4,374 shortcomings were cited, out of which 39 word groups were identified for qualities and 33 for shortcomings (Figure 1). The top five qualities of a *great surgeon* were dexterity (cited by 54% of respondents), meticulousness (18%), empathy (18%), communication skills with patients (16%), and listening skills with patients (16%). The top five shortcomings were arrogance (39% of respondents), temperamental (31%), clumsiness (17%), impatience (14%), and bullying (13%) (Tables 1, 2).

**Table 1:** Detailed responses for the “Great Surgeon”.

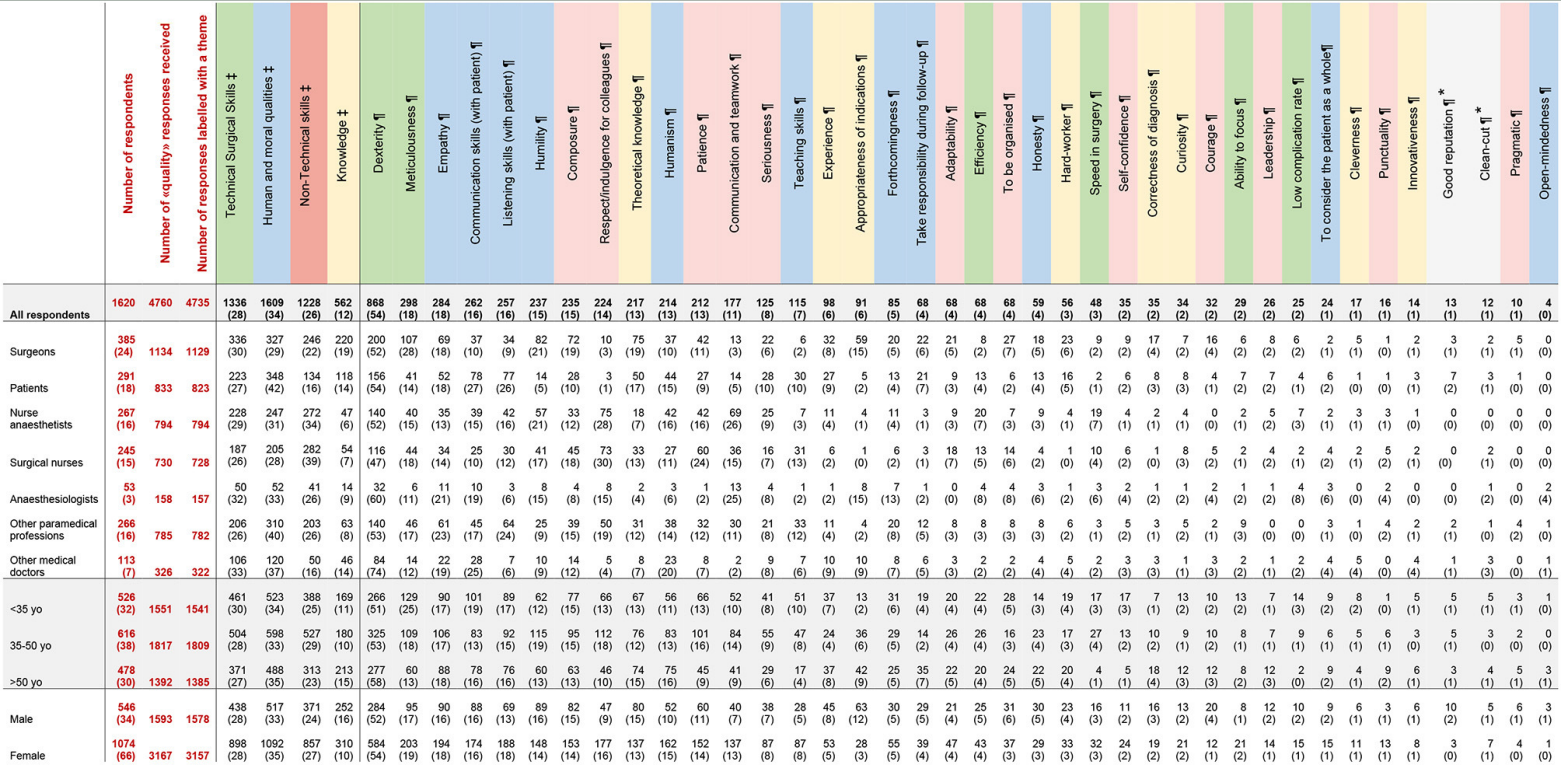


^‡^*n* (share of the total responses).

^¶^*n* (share of the total respondents).

^*^No theme was labeled to these qualities; Yo, years old.

**Table 2:** Detailed responses for the “Bad Surgeon”.

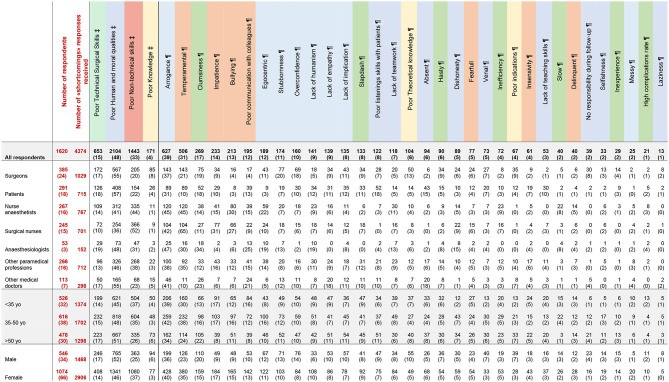


^‡^*n* (share of the total responses).

^¶^*n* (share of the total respondents). Yo, years old.

**Figure 2:**
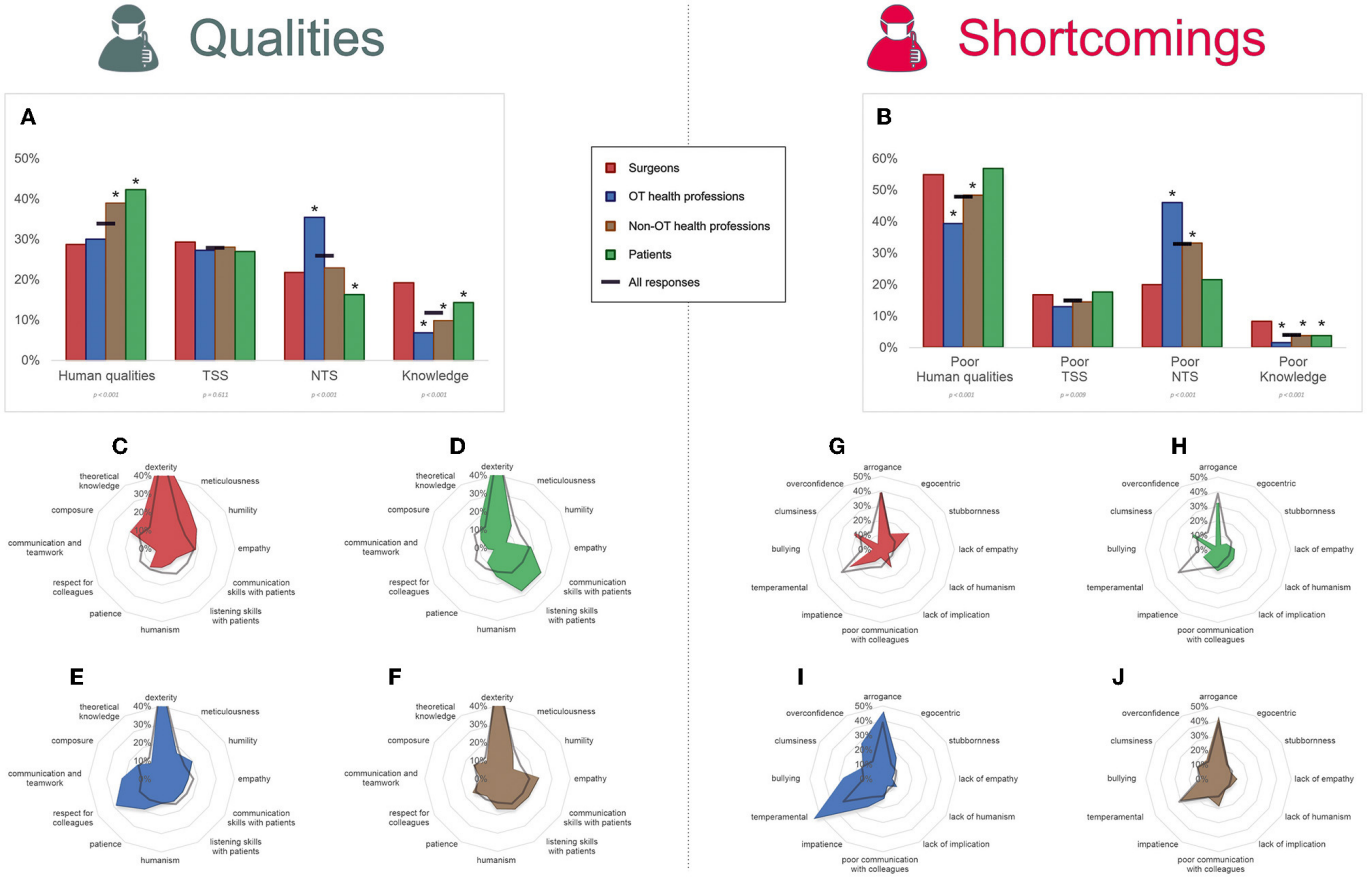
Qualities and shortcomings of surgeons, patients, and health professionals. Health professionals were divided into two groups: operating theater (OT) professionals and non-OT professionals, and their responses were compared to surgeons' and patients' responses. Qualities **(A)** and shortcomings **(B)** were spread into four main themes: human and moral qualities, technical surgical skills (TSS), non-technical skills (NTS), and medical knowledge. Graphs represent the proportion of responses belonging to the themes. Below each theme, the statistical significance of differences between groups (χ2 test) is indicated. In the graphs, asterisks indicate the groups which had significantly different answers compared to surgeons (Marascuilo procedure). Radar graphs show how are distributed the first 12 characteristics (in proportion of respondents who have given those characteristics). Radar graphs **(C–F)** show qualities for surgeons, patients, OT health professionals, and non-OT professionals, respectively. Radar graphs **(G–J)** show shortcomings for surgeons, patients, OT health professionals, and non-OT professionals, respectively.

A theme could be labeled for 4,735 of the 4,760 qualities, of which 34% (1,609) were in relation to human and moral qualities, 28% (1,336) to TSS, 26% (1,228) to NTS, and 12% (562) to knowledge (Figure 2). All 4,374 shortcomings could be labeled, of which 48% (2,016) were related to human and moral qualities, 33% (1,443) to NTS, 15% (654) to TSS, and 4% (171) to knowledge.

### Analysis of themes according to professional categories

Compared with surgeons (Figure 2), non-OT health professionals and patients put more emphasis on human and moral qualities (29 vs. 39% and 42%, respectively, *p* < 0.001). OT health professionals gave more importance to NTS than surgeons (35 vs. 22%, *p* < 0.001). Finally, knowledge was also more important for surgeons (19%) than any other professional categories (*p* < 0.001).

Considering shortcomings, poor TSS was similarly expressed in all professional categories' responses. Thirty-nine percent of OT health professionals and 49% of non-OT health professionals' responses referred to poor human qualities compared with 55% of surgeons' (*p* < 0.001). In contrast, 46% of OT health professionals' and 33% of non-OT health professionals' responses emphasized poor NTS as compared to only 20% of surgeons' (*p* < 0.001). Surgeons' responses referred more to poor knowledge than any other professional category (*p* < 0.001).

### Analysis of qualities and shortcomings according to profession

**Figure 3:**
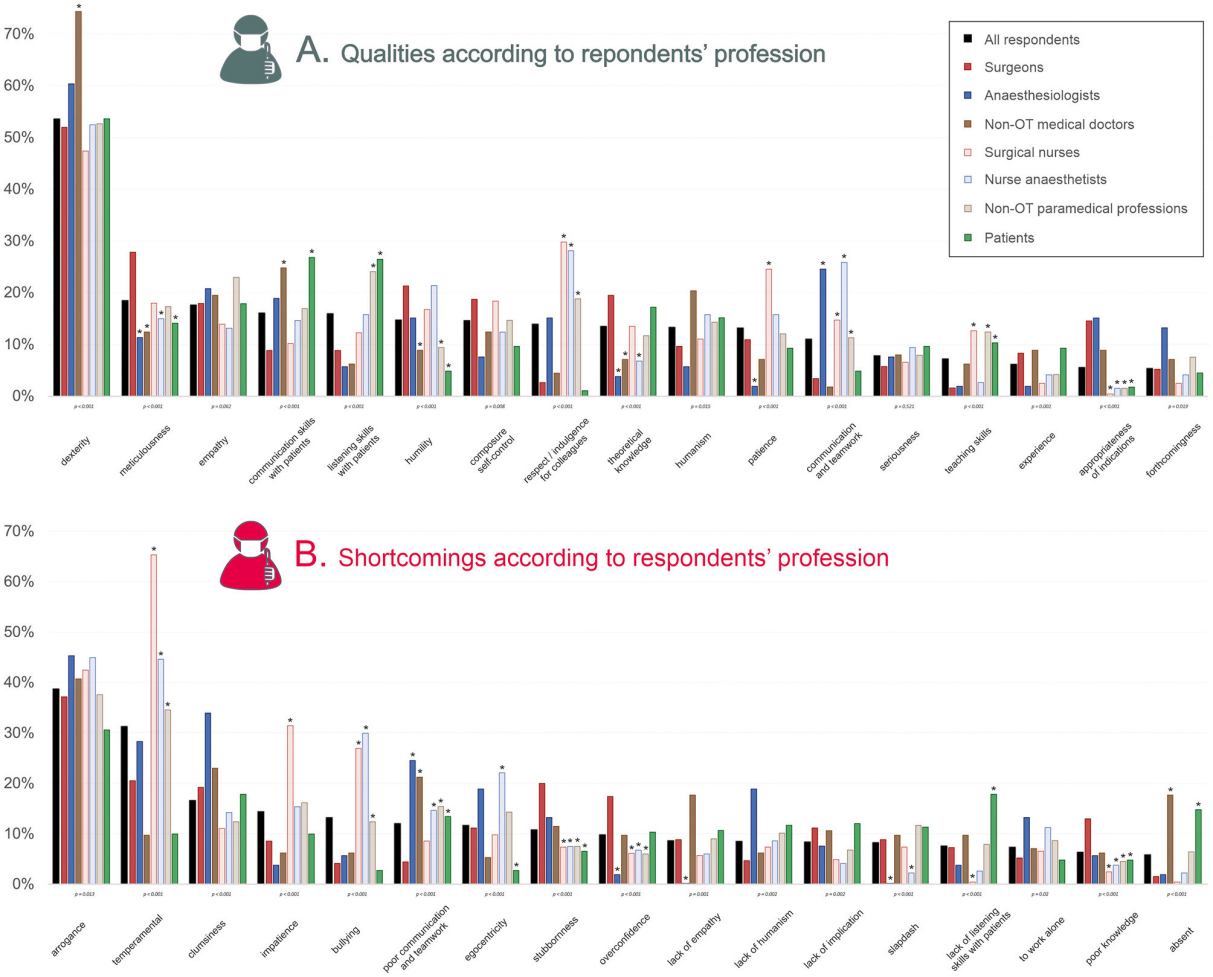
Qualities and shortcomings according to respondents' profession. The percentage of respondents having given the response is shown, for qualities **(A)** and shortcomings **(B)**. They are organized in a decreasing order based on the ≪ all respondents ≫ category. Are shown only qualities which were given by >10% of overall respondents; and shortcomings which were given by >5% of overall respondents. Below each characteristic, the statistical significance of differences between professions is shown (χ2 or Fisher's exact test when appropriate). In the graphs, asterisks indicate the professions which had significantly different answers compared to surgeons (Marascuilo procedure). OT, operating theater.

Notable differences compared to surgeons (Figure 3) were that communication, listening skills with patients and teaching skills were more cited by patients (*p* < 0.001). Respect, communication, and teamwork were more cited by anesthesiologists, surgical nurses, nurse anesthetics, and non-OT paramedical professionals (*p* < 0.001). Interestingly, the appropriateness of surgical indication was more cited by surgeons than by all paramedical professions and patients (*p* < 0.001). Concerning shortcomings, surgeons particularly insisted on stubbornness and overconfidence (*p* < 0.001) while temperamental and bullying traits were put forward by paramedical professionals (*p* < 0.001).

### Predictive factors of themes (qualities) according to demographic data

Multivariate analysis (Table 3) revealed that surgical nurses [OR 0.753 (0.598–0.947) *p* = 0.016] and non-OT paramedical professions [OR 0.776 (0.621–0.967) *p* = 0.024] were less concerned by TSS than surgeons. Nurse anesthetists [OR 1.901 (1.529–2.364) *p* < 0.0001], surgical nurses [OR 2.357 (1.875–2.967) *p* < 0.0001] and non-OT paramedical professionals [OR 1.313 (1.040–1.658) *p* = 0.022] were more concerned by NTS than surgeons, while patients [OR 0.726 (0.571–0.921) *p* = 0.009] and medical doctors [OR 0.670 (0.475–0.931) *p* = 0.020] were less. Non-OT medical doctors [OR 1.459 (1.120–1.895) *p* = 0.005], non-OT paramedical professions [OR 1.592 (1.291–1.964) *p* < 0.001], and patients [OR 1.776 (1.462–2.160) *p* < 0.0001] put more emphasis on human and moral qualities than surgeons. Finally concerning knowledge, the older respondents were more concerned by this theme than younger ones [OR 1.307 (1.047–1.633) *p* = 0.018]. Also, anesthesiologists [OR 0.445 (0.240–0.764) *p* = 0.006], nurse anesthetics [OR 0.290 (0.203–0.405) *p* < 0.0001], surgical nurses [OR 0.373 (0.264–0.521) *p* < 0.0001], non-OT paramedical professions [OR 0.403 (0.291–0.553) *p* < 0.0001], and patients [OR 0.713 (0.551–0.918) *p* = 0.009] mentioned less knowledge than surgeons.

**Table 3:** Predictors of responses.

		**Responses**		**Univariate analysis**		**Multivariate analysis**	
		**Yes (%)**	**No (%)**	**OR**	***p***	**OR (IC 95%)**	***p***
Technical surgical skills	Overall	1,336 (28)	3,399 (72)				
	Age				0.157		
	< 35 yo	461 (30)	1,080 (70)				
	35–50 yo	504 (28)	1,305 (72)			0.918 (0.788–1.069)	0.271
	>50 yo	371 (27)	1,014 (73)			0.866 (0.735–1.020)	0.086
	Professional category				0.119		
	Surgeons	336 (30)	793 (70)				
	Other medical doctors	106 (33)	216 (67)			1.103 (0.841–1.440)	0.475
	Anesthesiologists	50 (32)	107 (68)			1.040 (0.718–1.489)	0.834
	Nurse anesthetists	228 (29)	566 (71)			0.893 (0.722–1.104)	0.297
	Surgical nurses	187 (26)	541 (74)			0.753 (0.598–0.947)	**0.016** ^*^
	Other paramedical professions	206 (26)	576 (74)			0.776 (0.621–0.967)	**0.024** ^*^
	Patients	223 (27)	600 (73)			0.843 (0.685–1.036)	0.105
	**Sex**						
	Female	898 (28)	2,259 (72)				
	Male	438 (28)	1,140 (72)	0.967 (0.845–1.106)	0.62	0.883 (0.758–1.028)	0.111
Non-technical skills	Overall	1228 (26)	3,507 (74)				
	Age				**0.0001** ^***^		
	< 35 yo	388 (25)	1,153 (75)				
	35–50 yo	527 (29)	1,282 (71)			1.041 (0.889–1.220)	0.616
	>50 yo	313 (23)	1,072 (77)			0.915 (0.768–1.090)	0.321
	Professional category				**< 0.0001** ^***^		
	Surgeons	246 (22)	883 (78)				
	Other medical doctors	50 (16)	272 (84)			0.670 (0.475–0.931)	**0.020** ^*^
	Anesthesiologists	41 (26)	116 (74)			1.286 (0.864–1.882)	0.204
	Nurse anesthetists	272 (34)	522 (66)			1.901 (1.529–2.364)	**< 0.0001** ^***^
	Surgical nurses	282 (39)	446 (61)			2.357 (1.875–2.967)	**< 0.0001** ^***^
	Other paramedical professions	203 (42)	279 (58)			1.313 (1.040–1.658)	**0.022** ^*^
	Patients	134 (16)	689 (84)			0.726 (0.571–0.921)	**0.009** ^**^
	**Sex**						
	Female	857 (27)	2,300 (73)				
	Male	371 (24)	1,207 (76)	0.825 (0.717–0.950)	**0.007** ^**^	1.105 (0.938–1.300)	0.236
Human and moral qualities	Overall	1609 (34)	3,126 (66)				
	Age				0.436		
	< 35 yo	523 (34)	1,018 (66)				
	35–50 yo	598 (33)	1,211 (67)			1.032 (0.891–1.196)	0.672
	>50 yo	488 (35)	897 (65)			1.065 (0.912–1.245)	0.426
	Professional category				**< 0.0001** ^***^		
	Surgeons	327 (29)	802 (71)				
	Other medical doctors	120 (37)	202 (63)			1.459 (1.120–1.895)	**0.005** ^***^
	Anesthesiologists	52 (33)	105 (67)			1.210 (0.838–1.727)	0.302
	Nurse anesthetists	247 (31)	547 (69)			1.098 (0.890–1.354)	0.382
	Surgical nurses	205 (28)	523 (72)			0.946 (0.755–1.185)	0.630
	Other paramedical professions	310 (40)	472 (60)			1.592 (1.291–1.964)	**< 0.0001** ^***^
	Patients	348 (42)	475 (58)			1.776 (1.462–2.160)	**< 0.0001** ^***^
	**Sex**						
	Female	1,092 (35)	2,065 (65)				
	Male	517 (33)	1,061 (67)	0.922 (0.811–1.047)	0.211	0.968 (0.836–1.119)	0.658
Knowledge	Overall	562 (12)	4,173 (88)				
	Age				**< 0.0001** ^***^		
	< 35 yo	169 (11)	1,372 (89)				
	35–50 yo	180 (10)	1,629 (90)			1.016 (0.810–1.277)	0.888
	>50 yo	213 (15)	1,172 (85)			1.307 (1.047–1.633)	**0.018** ^*^
	Professional category				**< 0.0001** ^***^		
	Surgeons	220 (19)	909 (81)				
	Other medical doctors	46 (14)	276 (86)			0.731 (0.510–1.029)	0.079
	Anesthesiologists	14 (9)	143 (91)			0.445 (0.240–0.764)	**0.006** ^**^
	Nurse anesthetists	47 (6)	747 (94)			0.290 (0.203–0.405)	**< 0.0001** ^***^
	Surgical nurses	54 (7)	674 (93)			0.373 (0.264–0.521)	**< 0.0001** ^***^
	Other paramedical professions	63 (8)	719 (92)			0.403 (0.291–0.553)	**< 0.0001** ^***^
	Patients	118 (14)	705 (86)			0.713 (0.551–0.918)	**0.009** ^**^
	**Sex**						
	Female	310 (10)	2,847 (90)				
	Male	252 (16)	1,326 (84)	1.746 (1.460–2.087)	**< 0.0001** ^***^	1.137 (0.925–1.396)	0.222

Results of univariate and multivariate analyses for the four main themes: human and moral characteristics, technical surgical skills, non-technical skills, and medical knowledge. The following variables were analyzed: age, sex, and professional category. *p* < 0.05^*^, *p* < 0.01^**^, *p* < 0.001^***^. Yo, years old. Statistically significant results: ^*^p < 0.05, ^**^p < 0.01. Bold values are statistically significant values, with *p* < 0.05.

### Analysis of the surgeons' subgroup

Three hundred and eighty-five surgeons (262 males and 123 females) from 12 different specialties participated in the survey. The mean age was 46 ± 15 years (range 25–84 y); 117 < 35 y, 121 between 35 and 50 y, and 147 > 50 y. Type of practice varied: 94 junior surgeons, 121 attending surgeons in academic centers, 49 attending in non-academic centers, and 121 in private practice.

**Figure 4:**
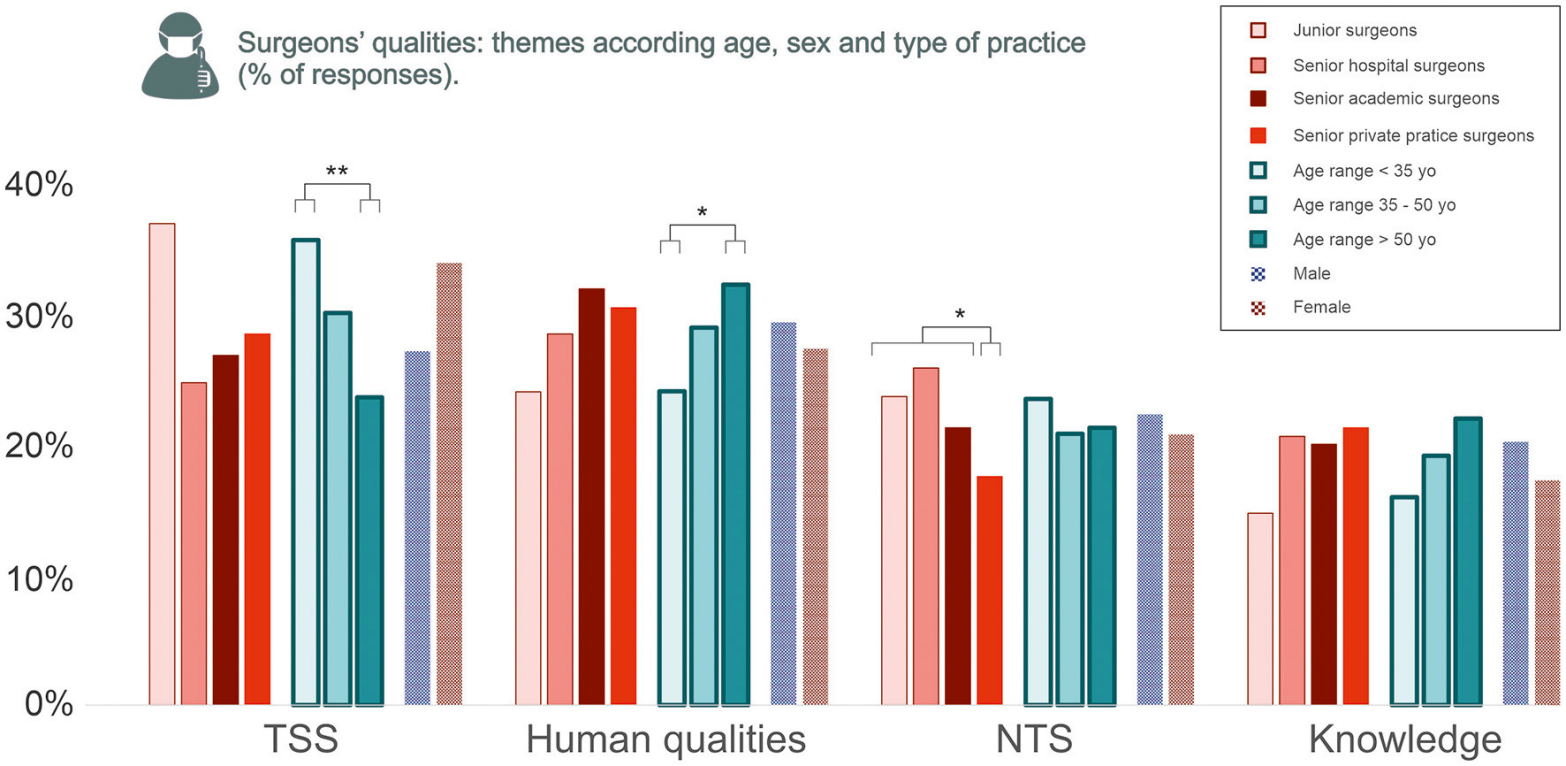
Surgeons' qualities: themes according to age, sex, and type of practice. The percentage of responses per theme are shown. The statistical significance of multivariate analysis is shown for each theme (non-significant not shown), using logistic regression (comparing age, private and public practice, and sex). NTS, non-technical skills; TSS, technical surgical skills. Statistically significant results: *p < 0.05, **p < 0.01.

TTS was less important for older surgeons [OR 0.560 (0.400–0.781) *p* = 0.007] as compared to younger surgeons. Human and moral qualities were put forward by the older surgeons [OR 1.518(1.083–2.137) *p* = 0.016]. NTS was less mentioned by surgeons in private practice [OR 0.676 (0.481–0.942) *p* = 0.023] as compared to those in public practice (Table 4; Figure 4).

**Table 4:** Predictors of responses for surgeons.

		**Responses**		**Univariate analysis**		**Multivariate analysis**	
		**Yes (%)**	**No (%)**	**OR**	***p***	**OR (IC 95%)**	***p***
Technical surgical skills	Overall	336 (30)	793 (70)				
	Age				**0.0008** ^***^		
	< 35 yo	125 (36)	220 (64)				
	35–50 yo	109 (30)	247 (70)			0.765 (0.552–1.058)	0.106
	>50 yo	102 (24)	326 (76)			0.560 (0.400–0.781)	**0.0007** ^***^
	**Sector of activity**						
	Public	233 (30)	548 (70)				
	Private	103 (30)	245 (70)	0.989 (0.751–1.299)	0.936	1.171 (0.871–1.570)	0.293
	**Sex**						
	Female	125 (34)	241 (66)				
	Male	211 (28)	552 (72)	0.737 (0.564–0.961)	**0.025** ^*^	0.825 (0.622–1.096)	0.183
Non-technical skills	Overall	246 (22)	873 (78)				
	Age				0.651		
	< 35 yo	81 (23)	264 (77)				
	35–50 yo	74 (21)	282 (79)			0.919 (0.636–1.327)	0.653
	>50 yo	91 (21)	337 (79)			0.922 (0.641–1.326)	0.659
	**Sector of activity**						
	Public	185 (24)	596 (76)				
	Private	61 (18)	287 (82)	0.685 (0.493–0.944)	**0.021** ^*^	0.676 (0.481–0.942)	**0.023** ^*^
	**Sex**						
	Female	76 (21)	290 (79)				
	Male	170 (22)	593 (78)	1.094 (0.807–1.479)	0.564	1.197 (0.870–1.657)	0.274
Human and moral qualities	Overall	327 (29)	802 (71)				
	Age				**0.031** ^*^		
	< 35 yo	83 (24)	262 (76)				
	35–50 yo	104 (29)	252 (71)			1.287 (0.911–1.821)	0.153
	>50 yo	140 (33)	288 (67)			1.518 (1.083–2.137)	**0.016** ^*^
	**Sector of activity**						
	Public	219 (28)	562 (72)				
	Private	108 (31)	240 (69)	1.155 (0.880–1.516)	0.306	1.055 (0.789–1.407)	0.714
	**Sex**						
	Female	101 (28)	265 (72)				
	Male	226 (30)	537 (70)	1.104 (0.840–1.457)	0.483	0.961 (0.737–1.324)	0.926
Knowledge	Overall	220 (19)	909 (81)				
	Age				0.115		
	< 35 yo	56 (16)	289 (84)				
	35–50 yo	69 (19)	287 (81)			1.185 (0.795–1.772)	0.405
	>50 yo	95 (22)	333 (78)			1.380 (0.938–2.045)	0.105
	**Sector of activity**						
	Public	144 (18)	637 (82)				
	Private	76 (22)	272 (78)	1.236 (0.902–1.683)	0.183	1.135 (0.817–1.571)	0.446
	**Sex**						
	Female	64 (17)	302 (83)				
	Male	156 (20)	607 (80)	1.213 (0.877–1.682)	0.240	1.092 (0.781–1.540)	0.610

Results of univariate and multivariate analysis in the surgeon's group for the four main themes: human and moral characteristics, technical surgical skills, non-technical skills, and medical knowledge. The following variables were analyzed: age, sex, main activity.

*p* < 0.05^*^, *p* < 0.01^**^, *p* < 0.001^***^. Yo, years old. Bold values are statistically significant values, with *p* < 0.05.

## Discussion

This survey is the first to deliver a multifaceted definition of a *great surgeon* by means of a large anonymous online survey involving multiple health professionals and patients. It shows that even if dexterity is a major quality, human qualities are of paramount importance. Knowledge seems to be underestimated by non-surgeons, although this quality seems essential to understand the disease and preparing the patient and OT team for the procedure.

We used an open questionnaire to not influence the answers. The methodological drawback was the need to classify answers into word groups and themes, which was done collectively to limit bias. We rapidly collected a vast number of enthusiastic responses especially from surgical nurses and nurse anesthetists, underlining the importance of this question in their workplace relationship.

The main quality by far is dexterity, a technical skill that remains true to the millennial-old etymology of the word “surgeon” in most civilizations: *surgery* in English and *chirurgie* in French both have the same Greek origin χε*ι*ρoυργ*ι*α (*kheirougia)* meaning “handiwork,” from *kheir* “hand” and *ergon “*work.” In another millennial-old culture, the same can be said of the Chinese word for surgery 手术 (*shoushù*) meaning the “skill” or “art” 术 of the “hand” 手. The essence of what makes a *great surgeon* seems unanimous and perpetually so, as someone with outstanding hand skills. As such, how may robotic surgery not only challenge the future of surgical procedures but also the essence of being a *great surgeon*?

Conversely, knowledge, and in a larger sense surgical wisdom, seems clearly underestimated by patients and all non-surgeon healthcare professions (especially anesthesiologists and non-OT medical doctors), which emphasizes the fact that surgery is perceived as essentially a technical, non-cerebral skill. In many respects, this sends back the historical barber-surgeons' opposition to medical societies, but also the age-old philosophical opposition between practical and theoretical knowledge. This opposition is also noted in the surgeon's subgroup, where young surgeons are more focused on TSS, which is regularly assessed by their peers to complete their training (12, 13). Older surgeons, who have long-acquired and refined experience, seem to be more concerned with human and moral qualities as well as medical knowledge. Thus, we may circle back to “A good surgeon knows how to operate; a better surgeon knows when to operate but the best surgeon knows when not to operate”. Indeed, surgery should not be reduced to craftsmanship. Claude Bernard (1813–1878) insisted that observation, experience, and understanding of underlying disease mechanisms should be the chief concern of a *great surgeon*, who should gather knowledge while caring for the patient (14, 15). However, we should also be wary of the primacy of knowledge when the focus on scientific publications and communications supersedes time spent operating patients in the OT: International reputation does not make a *great surgeon*, and a distinction should be made with what could be expected from a “great academic surgeon” (16). Surgery also surpasses craftsmanship by its humanism (17). The patient must remain the centerpiece beyond any technical skill or scientific activity as for the patient, nothing can replace the benevolent and reassuring contact with the surgeon who is about to undertake a procedure (18). This is confirmed in our study, as from the patients' perspective, human and moral qualities were more mentioned than in any other group, especially communication and listening skills. Likewise, teaching skills should not be limited to academic surgeons teaching trainees, as nurses and patients were the main groups to insist on this trait: Surgeons are better when their work is explained and understood by others. These results should be key for surgeons as they are the reflection of the shift to shared decision-making and patient-centered care (19–21). The fact that surgeons underestimated this point as compared to patients shows there is still considerable room for improvement.

This survey shows that NTS is another area for improvement for surgeons. Indeed, NTS emerged as a clear priority for OT health professionals with qualities such as respect, indulgence, communication, and leadership. The OT is a mysterious place to the outside world, which may explain why NTS are scarcely mentioned by patients. The importance that OT health professionals place on NTS reflects the need for a *great surgeon* to demonstrate respect and leadership to colleagues in the OT (22). Professionalism training is a major issue to avoid the stereotype of the temperamental, impatient, and bullying surgeon with poor communication and teamwork (23–25). The fact that stubbornness and overconfidence (key characteristics of the “*God complex*”) were more mentioned by surgeons themselves is encouraging, emphasizing self-consciousness and desire to change (26).

Of course, expectations may vary according to surgical practice. For example, one might expect empathy, or human and moral qualities, to be more important in breast cancer surgery, whereas technical skill can be perceived as the main quality in functional mechanical surgery such as knee surgery for sportsmen. Our survey lacked statistical power to analyze this aspect, but focusing on differences between specialties would be interesting to decipher the complexity of the OT. Although gender in our pool of respondents was not evenly distributed and may be a limitation to the study (most female respondents were younger and from a paramedical profession, reflecting healthcare demographics in France), it must be noted that sex did not influence how a *great surgeon* was defined based on the multivariate analyses. From a different perspective, this reinforces previous gender studies that great male and female surgeons may have similar qualities and outcomes (27, 28).

By confronting perspectives of this multifaceted survey, our work raises the question of “who” can best judge what a *great surgeon* is. Apart from dexterity, which was mentioned by all participants, everyone answered in line with their own interests, according to their own interactions and expectations of a *great surgeon*. For example, the patient prioritized listening skills whereas the anesthesiologist praised teamwork. But would the anesthesiologist give the same response before going under the knife himself? Also, in the era of online ratings, is a “5-star surgeon” really a *great surgeon*? Should the surgeon try to please patients in order to obtain a good notation? A poorly trained, arrogant, and manipulative surgeon may easily obtain great ratings by focusing on charm and communication skills with patients. Conversely, many surgeons we met during our study insisted that the essence of a great surgeon should be the ability to treat patients, even if those patients do not actually perceive or understand the qualities: knowledge, appropriateness of indications, dexterity, and recognition and management of complications. More specifically, although management of complications or the related concept of failure to rescue (death of a patient after potentially treatable complications) was not emphasized in our study, they seem crucial because of the underlying integrity and honesty they require, a form of humanism that is invisible to the patient (29, 30).

Limits of our study include the fact that participation in the survey was voluntary, which could have been a selection bias of respondents. Also, the fact that males were underrepresented may be a bias, as increasing the number of male respondents could reveal gender differences. Our study was not designed to be representative of any specific population but rather to confront the perspectives of a large number of surgeons, health professionals, and patients. Thus, we deliberately confronted subjective points of view, as evermore so, expectations of the *great surgeon* are set subjectively by colleagues and patients.

In summary, a *great surgeon* is not only a skilled craftsman but combines a subtle mixture of human and moral qualities, NTS, and medical knowledge. Surgeons should be aware of the different work settings (with patients or with colleagues) which each require a different set of skills. Improvement action to reach the status of *great surgeon* requires spending time in the operating theater and online reading up-to-date literature to be at the top of the game. Even then, emotional intelligence and leadership skills are not a given and surgeons should be specifically trained throughout their career.

## Conclusion

Technical surgical skills (first and foremost dexterity) are essential and defining qualities for a *great surgeon*, for whom the hands are the main day-to-day tools and remain the symbol of an age-old profession for the surgeons themselves, their patients, and their healthcare colleagues. A *great surgeon*, however, requires more than that especially in a patient-centered era. Surgery should not be limited to a technique, and on the contrary, surgeons should embrace humanism, mainly by building considerate leaderships in the operating theater and trusting relationships with their patients. Lastly, knowledge, although undermined in our results, may be the key to becoming a *great surgeon*: to best adapt the technical skills to the patient's disease, anticipate and organize the surgical team, and reassure the patient by breaking down information according to their needs.

## Data availability statement

The raw data supporting the conclusions of this article will be made available by the authors, without undue reservation.

## Author contributions

FSim and RL lead the study, collected data, and wrote the paper. EM, FSis, and SW collected and analyzed data. YP, BF, TB, HJ, FD, and E-NG wrote discussion and reviewed entire manuscript. All authors contributed to the article and approved the submitted version.
